# Next generation sequencing reveals a novel nonsense mutation in *MSX1* gene related to oligodontia

**DOI:** 10.1371/journal.pone.0202989

**Published:** 2018-09-07

**Authors:** Ondřej Bonczek, Peter Bielik, Přemysl Krejčí, Tomáš Zeman, Lýdie Izakovičová-Hollá, Jana Šoukalová, Jiří Vaněk, Tereza Gerguri, Vladimir J. Balcar, Omar Šerý

**Affiliations:** 1 Laboratory of Neurobiology and Molecular Psychiatry, Department of Biochemistry, Faculty of Science, Masaryk University, Brno, Czech Republic; 2 Laboratory of Neurobiology and Pathological Physiology, Institute of Animal Physiology and Genetics, The Academy of Sciences of the Czech Republic, Brno, Czech Republic; 3 Institute of Dentistry and Oral Sciences, Faculty of Medicine and Dentistry, Palacký University, Olomouc, Czech Republic; 4 Clinic of Stomatology, Faculty of Medicine, Masaryk University and St. Anne’s University Hospital, Brno, Czech Republic; 5 Biomolecular Modelling Laboratory, The Francis Crick Institute, London, United Kingdom; 6 Laboratory of Neurochemistry, Bosch Institute and Discipline of Anatomy and Histology, School of Medical Sciences, Sydney Medical School, The University of Sydney, Sydney, New South Wales, Australia; German Cancer Research Center (DKFZ), GERMANY

## Abstract

Tooth agenesis is one of the most common craniofacial disorders in humans. More than 350 genes have been associated with teeth development. In this study, we enrolled 60 child patients (age 13 to 17) with various types of tooth agenesis. Whole gene sequences of *PAX9*, *MSX1*, *AXIN2*, *EDA*, *EDAR* and *WNT10a* genes were sequenced by next generation sequencing on the Illumina MiSeq platform. We found previously undescribed heterozygous nonsense mutation g.8177G>T (c.610G>T) in *MSX1* gene in one child. Mutation was verified by Sanger sequencing. Sequencing analysis was performed in other family members of the affected child. All family members carrying g.8177G>T mutation suffered from oligodontia (missing more than 6 teeth excluding third molars). Mutation g.8177G>T leads to a stop codon (p.E204X) and premature termination of Msx1 protein translation. Based on previous *in vitro* experiments on mutation disrupting function of Msx1 homeodomain, we assume that the heterozygous g.8177G>T nonsense mutation affects the amount and function of Msx1 protein and leads to tooth agenesis.

## Introduction

Tooth development (odontogenesis) is a highly complex process involving interplay between the oral ectoderm and the ectomesenchyme cells putatively derived from the neural crest [1]. Signals from the underlying signalling centres are mediated by more than 350 signalling molecules including transcription factors, growth factors, receptors and other regulation proteins [2]. Synergistic and antagonistic interactions between various peptides and protein molecules may lead to their local activation or inhibition. The final morphology, position and the number of teeth is fully and precisely determined by these complex signalling networks. It is crucial for the participating molecules to be in the right place at the right time and in an exact quantity [3,4]. Molecular biology methods, such as genome-wide gene expression analysis by microarray assays, revealed expression profiles of the developing dentition and have identified several genes with hitherto unknown functions as possible contributors to the mechanisms of tooth development [5]. The cost and speed of DNA and RNA sequencing have been significantly improved because of the invention of the new sequencing platforms and their continuous iterations to achieve higher efficiency [6]. Next Generation Sequencing (NGS) is one of the major platforms making the recent advances possible [7,8].

Congenitally missing teeth (CMT), also known as tooth/dental agenesis (“hypodontia” is not an accurate term in this context; see below) is one of the most prevalent multifactorial craniofacial defects found in 0.15% - 16.2% of population with a 1.37 times higher prevalence in females than in males [9,10]. According to the number of missing teeth, the dental agenesis can be classified as hypodontia (less than 6 missing teeth other than third molars), oligodontia (6 and more missing teeth other than third molars) while the complete absence of teeth is called anodontia [10].

Congenitally missing teeth can occur in an isolated form or in a relationship with some syndromes such as cleft lip, cleft palate (or both) [11], Down-, Rieger- and Book- syndromes [12], Witkop syndrome associated with *MSX1* gene mutations [13] or ectodermal dysplasia with the most common X-linked inheritance are caused by mutations in *EDA* gene (ectodysplasin), *EDAR* gene (ectodysplasin A-receptor) or *EDARADD* gene (*EDAR* associated death domain) [14]. The *MSX1* gene mutations are typified by autosomal-dominant isolated form of congenitally missing teeth but sporadic forms of congenitally missing teeth are also common variants (see Table 1). The sporadic forms are characterised by the presence of congenitally missing teeth either in a single proband and no other family member or in several members of a family but with no clearly defined pattern of inheritance.

**Table 1:** Summary of published mutations in MSX1 gene.

dbSNP rs# cluster id^1^	CDS^2^	Exon no.	Amino acid change^2^	Reference	Phenotype, notice^2^
None	c.62dupG	1	p.G22RfsX168	[15]	Autosomal dominant, non-syndromic, oligodontia
rs36059701	c.119C>G		p.A40G	[16,17]	No significant association
rs121913130	c.182T>A		p.M61K	[18]	Autosomal dominant tooth agenesis*
rs104893852	c.314C>A		p.S105X	[19]	Autosomal dominant tooth agenesis and orofacial clefting*
None	c.416G>A		p.W139X	[20]	Autosomal dominant, non-syndromic oligodontia*
None	c.453G>T	2	p.R151S	[21]	Autosomal dominant hypodontia*
None	c.476T>G		p.L159R	[22]	Autosomal dominant, non-syndromic, oligodontia
None	c.521C>T		p.T174I	[23]	Sporadic form of hypodontia*
None	c.526C>T		p.R176W	[24]	Autosomal dominant, non-syndromic, oligodontia
None	c.559C>T		p.Q187X	[25]	Autosomal dominant, non-syndromic, oligodontia*
None	c.565C>T		p.Q189X	[26]	Oligodontia, cleft lip*
None	c.572_573ins GCAAGTT		p.F191fs	[27]	Autosomal dominant, non-syndromic oligodontia
None	c.581C>T		p.A194V	[28]	Selective non-syndromic oligodontia*
None	c.587G>C		p.R196P	[29]	Autosomal dominant tooth agenesis*
None	c.605C>A		p.S202X	[30]	Witkop syndrome, oligodontia*
None	c.610G>T		p.E204X	This study	Non-syndromic oligodontia
None	c.614T>G		p.L205R	[23]	Autosomal dominant, non-syndromic oligodontia*
None	c.644insA		p.Q216QfsX125	[24]	Autosomal dominant, non-syndromic, oligodontia*
None	c.655G>A		p.A219T	[31]	Autosomal recessive oligodontia with dental anomalies*
None	c.662C>A		p.A221E	[32]	Autosomal dominant, non-syndromic, oligodontia*
None	c.665-666insA		p.N222KfsX118	[33]	Autosomal dominant, non-syndromic, oligodontia
None	c.671T>C		p.L224P	[34]	Autosomal dominant, non-syndromic hypodontia*
None	c.707delG		p.K237SfsX2	[33]	Autosomal dominant, non-syndromic, oligodontia
None	c.750_751insACCGGCTGCC		p.F251PfsX92	[35]	Autosomal dominant, non-syndromic hypodontia
rs515726227	c.910_911dupTA		p.X304YextX48	[36]	Autosomal dominant, non-syndromic, oligodontia

^1^According to NCBI dbSNP Short Genetic Variations: geneID: 4487

^2^Data and localizations were obtained from original articles

*Many of the reviewed articles used what would be an incorrect numbering of exons of the MSX1 gene according to the current reference sequences from NCBI database. The “wrong” numbering is marked with asterisk.

The *MSX1* gene is expressed in dental mesenchyme and plays an important role during reciprocal epithelial-mesenchymal interactions. The Msx1^-^/ Msx1^-^ deficient mice exhibit complete secondary cleft lip palate and abnormality in tooth development. The development of the tooth is arrested at the bud stage [37,38].

**Fig 1:**
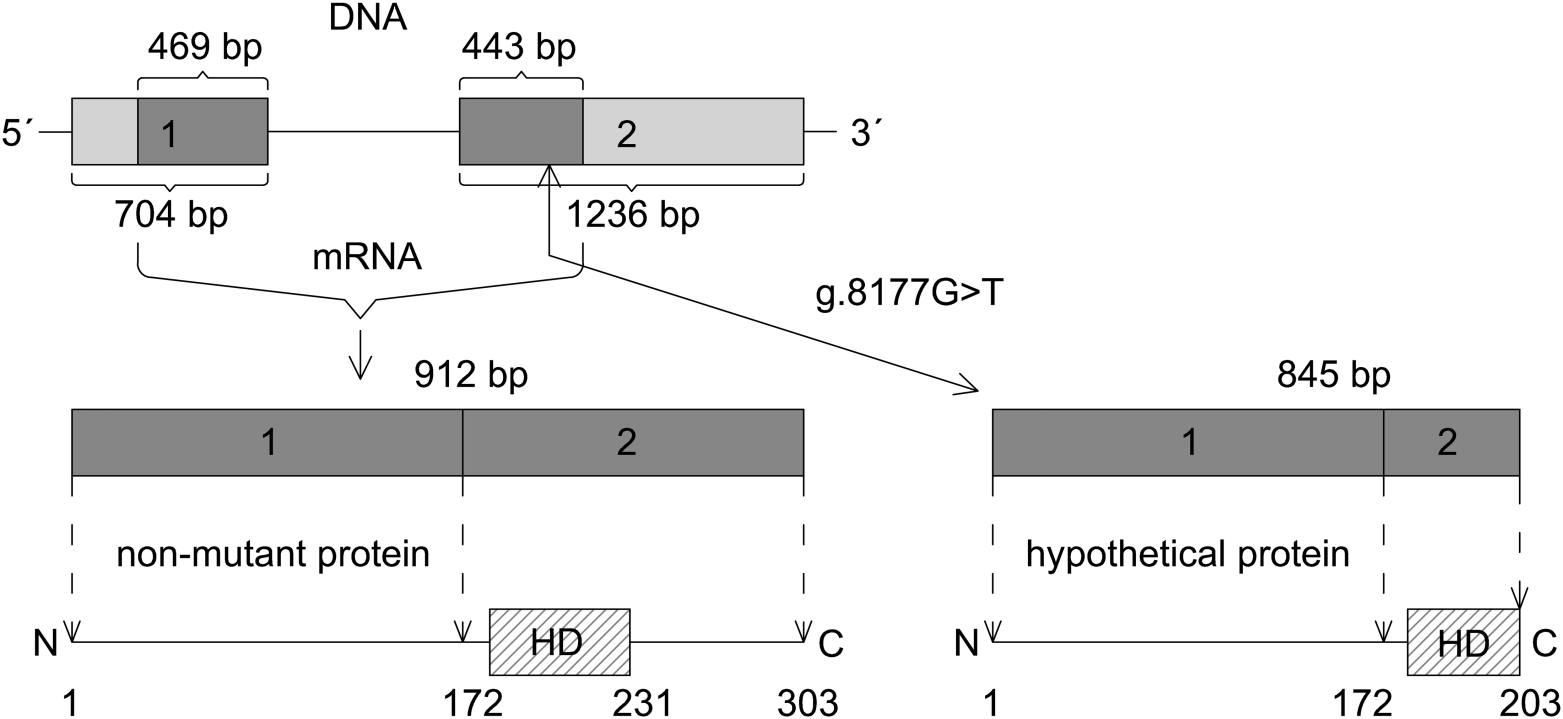
Schematic description of nucleotide sequence, mRNA and protein structure of the MSX1. Light grey colour shows untranslated region (UTR); dark grey colour shows coding sequence (CDS); HD = homeodomain. The hypothetical mutated protein product could be 100 amino acids shorter (203 amino acids) than non-mutant variant of the Msx1 (303 amino acids).

In humans, *MSX1* gene is located on the short arm of chromosome 4 (4p16.2). The structure of the gene consists of 2 exons. Both exons represent UTR´s regions (5´- and 3´- end) and contain 912 bases (including the stop codon) encoding 303 amino acids constituting the human Msx1 protein. The most interesting protein element–the homeodomain—is encoded in the second exon (Fig 1).

The aim of the present study was to screen *PAX9*, *MSX1*, *AXIN2*, *EDA*, *EDAR and WNT10a* genes, using the technique of whole gene DNA sequencing and DNA collected from a group of child patients with oligodontia, in the hope of identifying a mutation which could constitute a possible genetic basis of the patients’ phenotype. This resulted in the identification of a novel SNP in the *MSX1* gene as the most likely genetic correlate of the oligodontia. Based on our previous experience with this type of approach [16], we then opted for a family study to further test the relationship between the mutation and the phenotype.

## Materials and methods

For the screening of DNA mutations by next generation sequencing (NGS); a total of 60 DNA samples of patients aged between 13 and 17 years with various types of tooth agenesis were obtained at the Clinic of Stomatology of St. Anne’s Faculty Hospital in Brno and at the Institute of Dentistry and Oral Sciences in Olomouc.

Dental phenotypes were assessed by oral examinations and inspections of dental radiographs obtained by Planmeca ProMax 3D (Planmeca Oy Finland) using the Planmeca Romexis software (version 2.9.2). Complete dentition was defined as 32 permanent teeth (including the third molars). Oral panoramic radiographs (Planmeca ProMax 3D, Planmeca Oy Finland) were taken in the patient with mutation g.8177G>T (c.610G>T) and members of his family (samples Z606B, Z606C, Z606D, Z606E, Z606F). The study protocol and informed consents (No: Fm-L009-001-ZUBNI-014) were reviewed and approved by ethical committees of Faculty of Medicine and Dentistry, Olomouc and Faculty of Medicine, Brno. The study was performed in accordance with the Declaration of Helsinki. All participants in this study gave their written informed consent.

200 μL of whole blood were taken from the fingers by EDTA coated capillaries Microvette 200 μL K3 EDTA (Sarstedt, Germany). DNA was extracted from 200 μL whole blood by using automatic instrument ZEPHYRUS Magneto with modified protocol as specified for BodyFluid DNA/RNA isolation kit (Elisabeth Pharmacon, Czech Republic). The yield of genomic DNA was verified by spectrophotometric measurement. Size and quantity of DNA fragments were determined on 2200 TapeStation Instrument (Agilent Technologies, USA). Paired end sequencing libraries were constructed from 150 ng of fragmented DNA in 50 μL PCR water using the Covaris S220 Sonicator (Covaris, USA) to a size 200 bp (treatment time 180 s, volume 130 μL). Library preparation was performed by SeqCap EZ System (Roche NimbleGen, USA) with probes for *PAX9*, *MSX1*, *AXIN2*, *EDA*, *EDAR*, and *WNT10A* genes according to manufacturer’s recommendations. Twelve samples were mixed to one library. The concentration of captured library was determined through KAPA qPCR KAPA Library Quantification Kit (Kapa Biosystems, USA) to produce clusters suitable for the Illumina MiSeq platform (Illumina, USA). The library was sequenced on a MiSeq V2 2x151 bp sequencing run according to manufacturer recommendations for 14 pM libraries.

Obtained NGS data were analyzed according to Roche NimbleGen workflow [39]. Hg38 was used as the reference genome. BWA 0.7.13 software package [40] was used for indexing the reference genome and alignment of reads. Adapter trimming was performed using Trimmomatic 0.32 software [41]. PCR duplicates were removed using Picard Tools 1.110 software. Variant calling and filtering was performed using SAMtools 1.3 and BCFtools 1.3 software [42,43]. The depth of coverage was calculated for each position on the reference genome corresponding to the selected genes (*PAX9*, *MSX1*, *AXIN2*, *EDA*, *EDAR*, and *WNT10A*) using software R [44]. Sequence data were browsed through using Integrative Genomics Viewer 2.3 (IGV) [45,46].

For control sequencing of the second exon of the *MSX1* gene and intron-exon boundaries, the automated ABI 3130 Genetic Analyzer was used. DNA sequence was amplified by PCR in a 20 μL reaction volume. Specific PCR method and sequencing primers were used as described previously [16].

PCR analysis of exon 2 of the *MSX1* gene was performed by the KAPA2G Robust HotStart kit (Kapa Biosystems, USA) + 5% DMSO and 1% 7-Deaza-dGTP (Roche Diagnostics, USA). Robust polymerase, DMSO and 7-Deaza-dGTP were used for PCR mixtures because of the very high content of G + C bases in the nucleotide structure of *MSX1* gene. The conditions for PCR were 95°C for 10 min activation/denaturation step, followed by 40 cycles of 95°C for 60 s, 58°C (with 20% ramp) for 30 s, 72°C for 60 s, with a final extension for 7 min. The Veriti thermal cycler (Applied Biosystems, USA) was used for all PCR reactions.

Amplicons were purified by ExoI-FastAP (Fermentas, USA). The mixtures were incubated at 37°C for 15 min and subsequently at 85°C for 15 min to inactivate enzymes. Sequencing was performed with BigDye Terminator v.3.1 (Life Technologies, USA). Sequencing reactions/the products were purified by EDTA/ethanol precipitation, resuspended in 10 μL Hi-Di Formamide (Life Technologies, USA), and sequenced on the automated ABI 3130 Genetic Analyzer.

Finally, sequences were compared with the standard sequence NG_008121.1 of the *MSX1* gene obtained from the GenBank database by MEGA 7 software.

### Molecular modelling

Both the wild type and truncated version (Table 2) of Msx1 homeodomain (residues 173–230 and 173–203, respectively) were prepared using homology modelling software MODELLER [47] based on PDB template 1IG7. Schematic representation was prepared via visual molecular dynamics software (VMD) [48], Schrodinger Maestro software (Schrodinger, L. Biologics Suite 2017–1) and PyMOL software (The PyMOL Molecular Graphics System, Version 2.0 Schrödinger, LLC). DNA fragment was superimposed with the prepared structures from PDB in PyMOL.

**Table 2:** Amino-acid sequence for non-mutant and mutant Msx1 homeodomain used for homology modelling.

***MSX-1 (residues 173–230)***	RKPRTPFTTAQLLALERKFRQKQYLSIAERAEFSSSLSLTETQVKIWFQNRRAKAKRL
***MSX-1 mutant (residue 173–203)***	RKPRTPFTTAQLLALERKFRQKQYLSIAERA

## Results

Using the technique of whole gene next generation sequencing (NGS), DNA samples collected from 60 child patients were sequenced for mutation in *PAX9*, *MSX1*, *AXIN2*, *EDA*, *EDAR and WNT10a* genes. More than 3000 polymorphisms were found in the screened genes. We then looked for mutations which could be putatively linked to the oligodontia phenotype. In one female proband, we found a mutation in the *MSX1* gene causing premature stop codon in the second exon of the gene thus resulting in a significantly shorter version of the protein. We found no other mutations in the *MSX1* gene in this patient or in the remaining probands in the group. Additional screening of the female proband’s DNA, particularly of the genes listed above, revealed no other mutations which would correlate with her phenotype. We then opted for a family study to further test the relationship between the newly identified mutation and the oligodontia phenotype.

**Fig 2:**
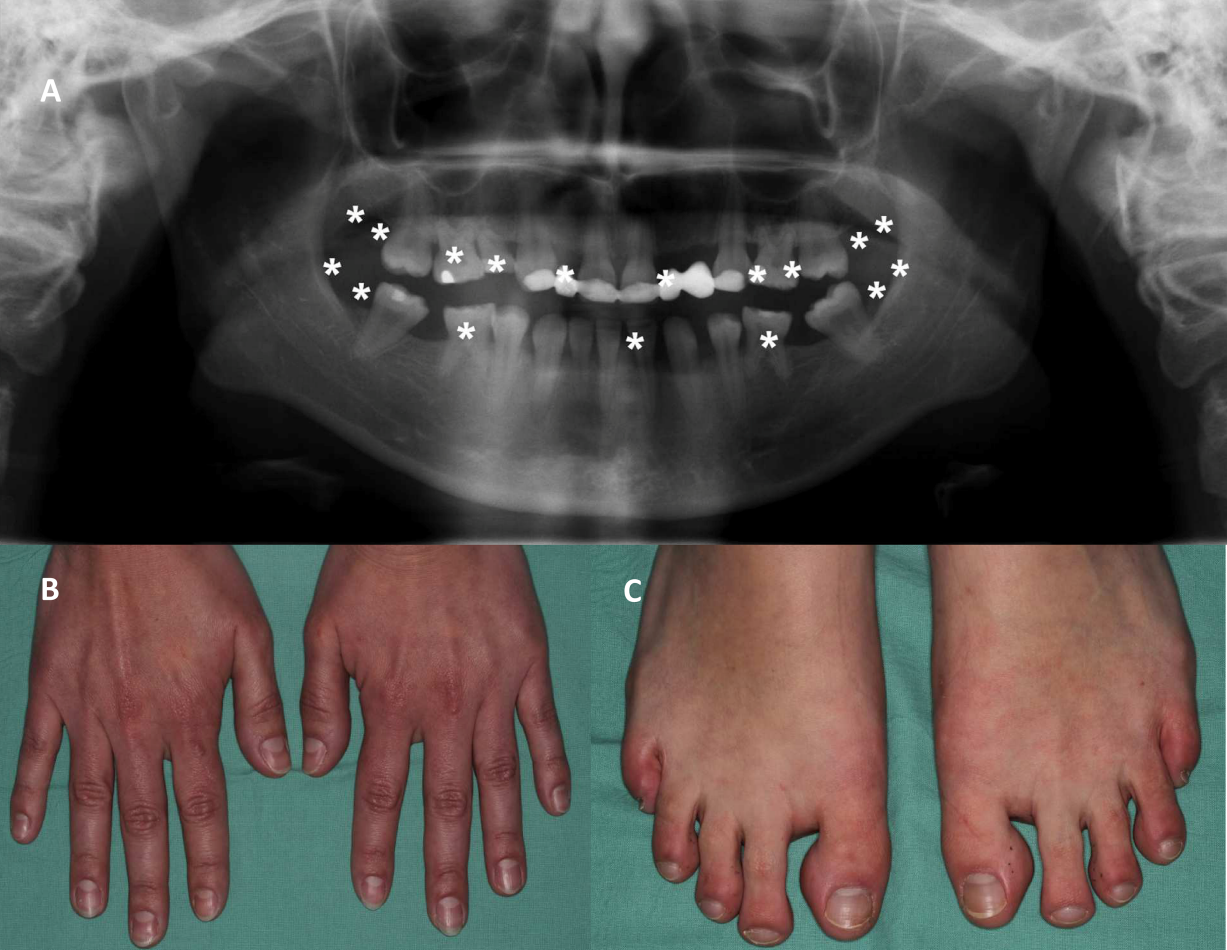
Proband´s tooth and nail phenotype. (A) Panoramic radiograph of proband Z606. This individual has a total 17 congenitally missing teeth (Agenesis 18, 17, 15, 14, 12, 22, 24, 25, 27, 28, 38, 37, 35, 31, 45, 47, 48). Condition after orthodontic and prosthetic treatment. (B) Nails on hands are in normal shape without any defect. (C) Nails on legs are in normal shape and without defects excepts the fifth finger nail that is hypoplastic.

The female proband (Z606) displayed oligodontia with 17 missing teeth (including third molars, see Fig 2A). To further characterize her phenotype, we excluded the presence of ectodermal dysplasia, Witkop syndrome (thick hair, normal development of eyebrows, eyelashes, nails–hands and feet—see Fig 2B and 2C), defective development of sweat glands and the presence of cleft lip/palate. In addition, using standard clinical genetic examination, we excluded other hereditary syndromes often present in patients with oligodontia.

**Fig 3:**
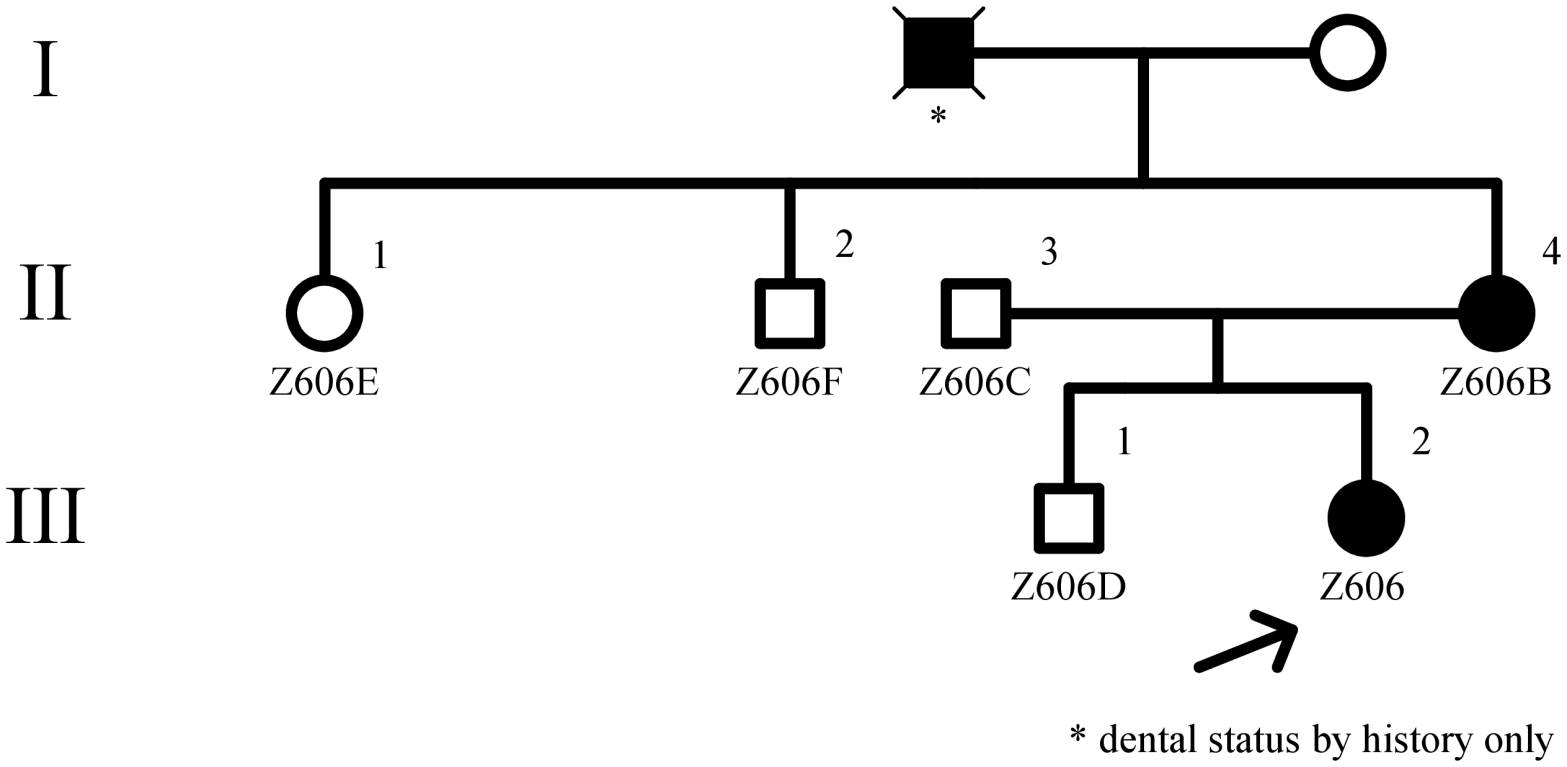
Pedigree of multigeneration family with oligodontia. The same g. 8177G>T mutation was observed only in family members with oligodontia (Z606 and Z606B) but not in unaffected relatives (Z606C, Z606D, Z606E, Z606F). The probands’ grandfather’s status was provided by family history only. The probands’ grandmother was unavailable for genotyping.

Genealogic examination revealed isolated oligodontia in the mother of the proband (Z606B) and in her father (information about father were obtained from family history only). The mother had no knowledge of other cases of oligodontia in the family. Two siblings of the mother (Z606E and Z606F) are healthy and have healthy children. An elder brother of the proband (Z606D) and the father (Z606C) have healthy developed dentition. The summary of all missing teeth and the reconstruction of the family pedigree are in Table 3 and in Fig 3 respectively.

**Table 3:** Summary of missing teeth in family with g.8177G>T nonsense mutation. Asterisks indicate the missing teeth. The column shows the sum of missing teeth, in parentheses missing teeth plus third molars are given (I…incisors, C…canines, P…premolars, M…molars).

Right quadrant									Left quadrant								
	M			P		C		I	I	C		P		M			
Upper	18	17	16	15	14	13	12	11	21	22	23	24	25	26	27	28	Total
Lower	48	47	46	45	44	43	42	41	31	32	33	34	35	36	37	38	
II:1 Z606E	*																0(1)
															
II:2 Z606F																	0(0)
															
II:3 Z606C																	0(0)
															
II:4 Z606B	*			*	*		*			*		*	*			*	9(13)
	*			*				*					*			*	
III:1 Z606D																	0(0)
															
III:2 Z606	*	*		*	*		*			*		*	*		*	*	13(17)
	*	*		*					*				*		*	*	

We then proceeded to collect DNA from five family members of Z606 proband and subjected the samples to capillary sequencing focusing on the second exon of the *MSX1* gene.

The mutation which we found earlier in Z606 and for which we were looking in her relatives, has not, to our knowledge, been previously reported in the literature. The heterozygous nucleotide change was identified in the second exon of the *MSX1*gene at the position g.8177G>T, using NG_008121.1 as the reference sequence (GenBank Database). The total average depth of coverage of analyzed sample was 98 and the mutated position was read 49 times; 17 times (35%) for G allele and 32 times (65%) for T allele. The nucleotide change produces the premature stop codon at the amino acid position 204 that would otherwise code for glutamic acid (p.204E -> p.204Stop). The triplet GAG is changed to TAG, or, in the case of mRNA, to UAG. The mutated protein product is 100 amino acids shorter than the non-mutant variant of the *MSX1*. The structures of both non-mutant and mutant variants of *MSX1* are shown in Fig 1.

**Fig 4:**
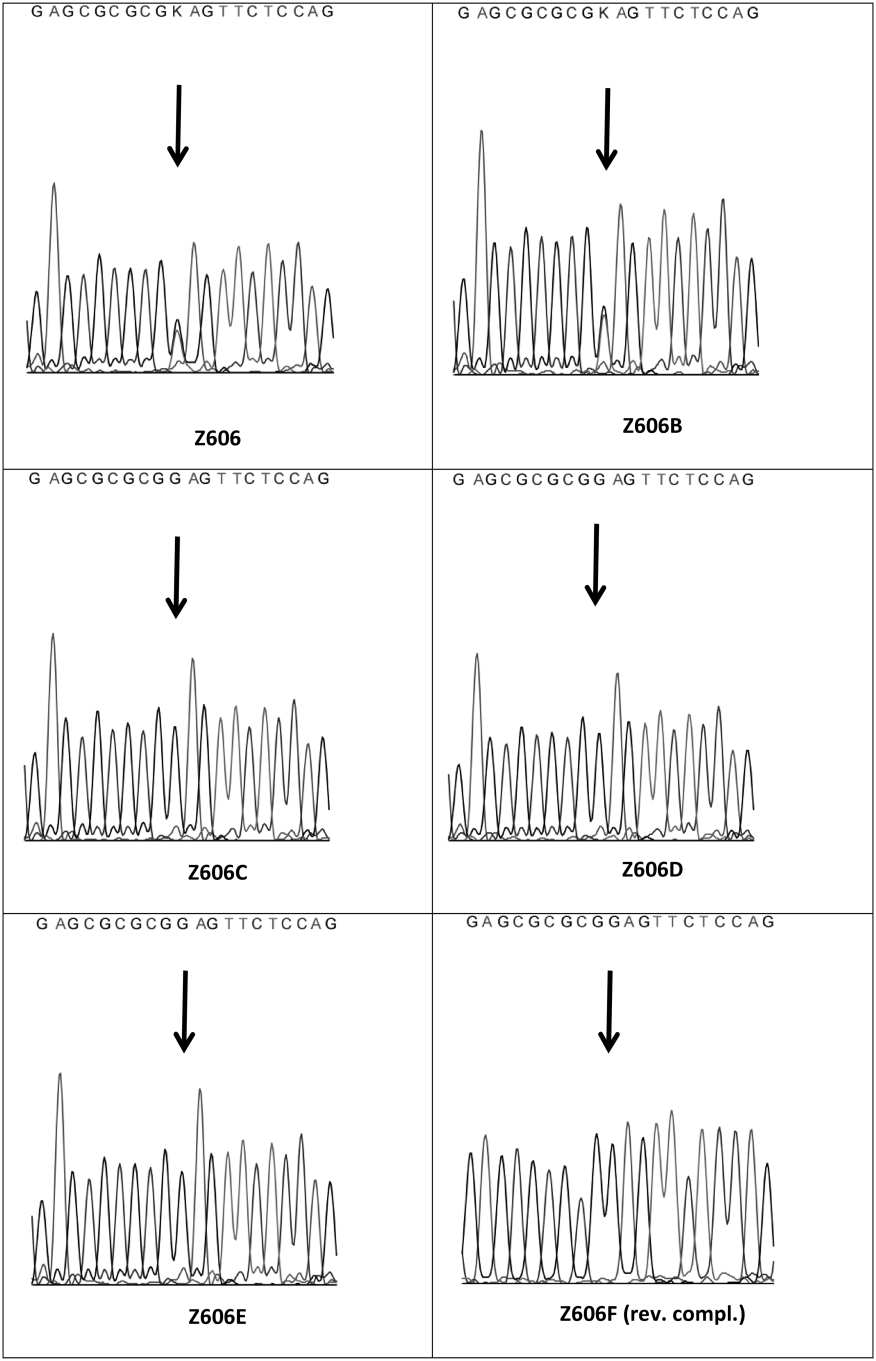
Chromatograms obtained from capillary sequencing of exon 2 in MSX1 gene. In samples Z606 and 606B is g.8177G>T mutation marked as “K” mixed base (G/T), in samples 606C, 606D, 606E, 606F G nucleotide in the 9527^th^ position of the MSX1 gene is indicated. In case of sample Z606F is chromatogram reverse complement.

We found the same heterozygous mutation g.8177G>T in one other family member (Z606B) with oligodontia. Mutation was not present in other family members who had complete sets of teeth (Z606C, Z606D, Z606E and Z606F). One additional mutation, g.8014_8022delT, was identified in the second exon of the *MSX1* gene in a sample collected from Z606F. This deletion was not associated with tooth agenesis [16]. This sample was further re-sequenced by a reverse primer normally intended for PCR amplification, the reason being that the deletion renders the use of the standard sequence primer (forward reading) unsuitable; indeed, its application lead to an unreadable chromatogram. The chromatograms from capillary sequencing are shown in Fig 4. No additional mutations were detected in *PAX9*, *AXIN2*, *EDA*, *EDAR* or *WNT10a* genes.

## Discussion

To date, over 30 mutations in *MSX1* gene have been related to tooth developmental diseases (see in Table 1). *MSX1* gene together with *PAX9* gene (recently reviewed by Bonczek *et al*, 2017 [49]) are two most studied genes related to tooth agenesis.

The heterozygous g.8177G>T substitution, identified for the first time in the present study, leads to premature stop codon and results in a protein sequence truncated to 203 amino acids compared to the normal protein with 303 amino acids. This novel mutation was identified neither in our previous capillary sequencing project screening 270 Czech individuals with tooth agenesis and 30 unrelated healthy control subjects [16] nor, to the best of our knowledge, in any other studies. There have been five other reports, though, of substitutions, each of them resulting in an appearance of the premature stop codon [19,20,25,26,30] (“see Table 1”).

Typically, the mutations in *MSX1* gene are associated with oligodontia inherited in the autosomal dominant manner; to date only one case of autosomal recessive oligodontia has appeared [31]. All these mutations are heterozygous and the missing teeth may result from haploinsufficiency of the Msx1 protein [50]. Affected individuals are missing a large number of teeth (mostly more than 10), [18,29,30]. This is also the case of the two family members identified in the present study who are missing third molars, second premolars, upper first molars, upper second incisors and one lower first incisor. The proband Z606 has more severe oligodontia, missing also second molars (together 17 teeth).

**Fig 5:**
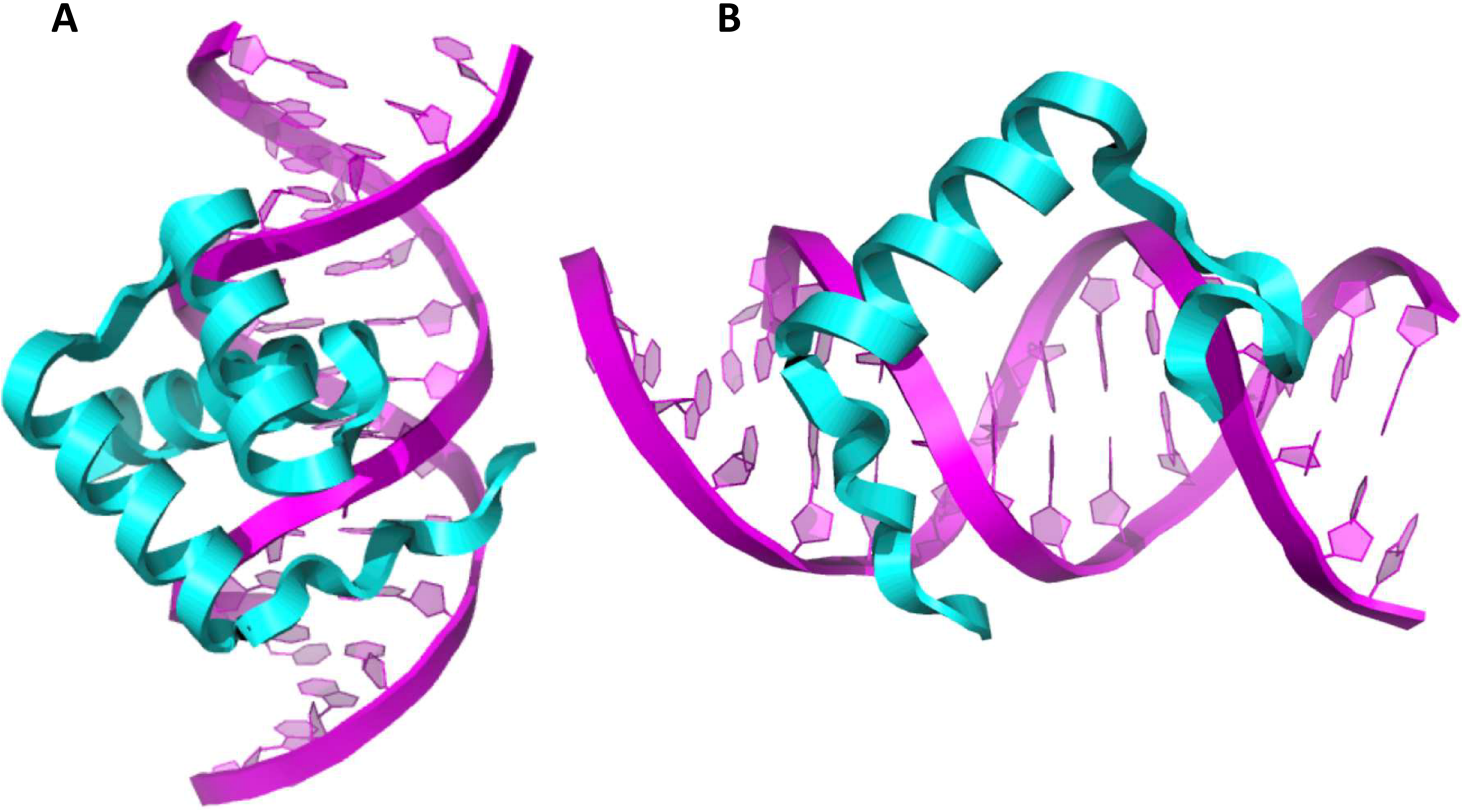
Msx1 homeodomains bound to DNA. (A) Non-mutant Msx1 homeodomain. (B) Mutant Msx1 homeodomain. Homeodomain is visualized as cartoon.

Most of the previously reported mutations (Table 1) were located in the highly conserved homeodomain (172^nd^-231^st^ amino acids) which is thought to play an important role in protein-protein interactions and DNA binding [51]. It has been known that an important factor in the protein stability is the presence of helix II [52] while helix III is important for DNA binding [53]. A protein-protein interaction between Pax9 and Msx1 has been demonstrated both *in vivo* and *in vitro* and it seems that this interaction is essential for the expression of *BMP4* in the mesenchyme [54]. Furthermore, Msx1 protein forms dimeric complexes with Dlx proteins through their homeo-domains; the binding being mediated by residues that are also required for their DNA binding activities [51]. The newly found heterozygous nonsense nucleotide change g.8177G>T is located in helix II of the homeodomain, thus probably affecting helix II and causing absence of helix III. Thus it seems that the polymorphisms affecting the homeodomain and associated with oligodontias, including the one in the present study, lead to amino acid changes that impact on the protein stability and/or the homeodomain’s DNA binding activity. In order to gain greater insight into the mechanism, we modelled the structures of homeodomains of both the non-mutant and mutant version of Msx1 (Fig 5A and 5B). As hypothesized, the structural modelling indeed revealed that the truncated version of Msx1 homeodomain lacks, at least in part, the ability to interact with DNA.

Cases of nucleotide changes leading to premature stop codon and having functional or morphological consequence have been described previously. For example, Liang *et al* (2012) [26] published heterozygous Q189X substitution in a Chinese family suffered from oligodontia and cleft lip. They performed relative-quantitative PCR and found out that mRNA expression level of the mutant *MSX1* was less than half of the non-mutant. Kimura *et al* (2014) [20] identified heterozygous nonsense c.416>A mutation producing W139X protein product in Japanese family with non-syndromic oligodontia. They carried out a functional expression study and reported no difference in the expression level between the mutant and non-mutant variants of Msx1, however, they noted a severe defect in the nuclear trafficking of the mutant protein.

In summary, we have found a heterozygous nonsense g.8177G>T mutation in *MSX1* gene which appears to be associated with severe tooth agenesis. Based on a review of previously published data complemented by the molecular modelling performed in the present study, we conclude that the newly identified mutation could influence specific properties of Msx1 protein thus possibly impairing its ability to interact with DNA and proteins. The mutation may therefore, produce a significant adverse effect on the role of Msx1 protein in the complex signaling mechanisms necessary for normal odontogenesis and result in oligodontia in the affected individuals.
